# Literature review on psychological interventions in school bullying: evaluating new trends and challenges

**DOI:** 10.3389/fpsyg.2025.1600785

**Published:** 2026-02-23

**Authors:** Davis Velarde-Camaqui, Luis Chunga-Pajares, Yrene Gloria Chamorro Bacilo, Karina Velarde-Camaqui

**Affiliations:** 1 Universidad César Vallejo, Herrera, Peru; 2 Institute for the Future of Education, Tecnologico de Monterrey, Monterrey, Mexico

**Keywords:** school bullying, psychological interventions, cyberbullying, systematic literature review (SLR), emotional well-being, educational innovation, higher education

## Abstract

School bullying remains a persistent challenge in educational settings, deeply affecting students’ emotional well-being and academic engagement. Although numerous psychological interventions have been implemented globally, disparities in effectiveness and adaptability persist across different contexts. However, existing literature lacks a comprehensive synthesis of how these interventions have evolved over the last decade and how they address emerging challenges such as cyberbullying and AI-driven aggression. This study aims to analyze the evolution, effectiveness, and adaptability of psychological interventions in school bullying based on a systematic literature review. A total of 21 open-access articles published between 2015 and 2024 were selected through Scopus, Web of Science, and SciELO using the PRISMA method and the Boolean formula “(program OR intervention) AND bull* AND (session).” The findings reveal that: interventions have progressed from behaviorist to systemic approaches; cognitive-behavioral and social–emotional learning models are the most effective; institutional policies significantly shape implementation success; interventions positively influence emotional well-being and academic outcomes; and current strategies remain insufficient to fully address new digital threats. In conclusion, (a) psychological interventions are effective when grounded in strong theoretical models; (b) emotional and academic outcomes improve with targeted strategies; (c) policies and institutional frameworks are critical for sustainable implementation; (d) cyberbullying and AI-related aggression require digital literacy components; and (e) future programs must integrate interdisciplinary tools to remain effective in evolving contexts.

## 1 Introduction

School bullying remains a deeply rooted issue in educational systems, directly affecting students’ emotional and psychological development. It is characterized as intentional, repetitive aggressive behavior involving a power imbalance, where a perpetrator seeks to dominate a victim through physical, verbal, social, or digital violence (Ahmed et al., 2022; Menesini and Salmivalli, 2017; Timmons-Mitchell et al., 2021). Psychological interventions have emerged as one of the most effective strategies to mitigate the impact of bullying, promoting student well-being and fostering a healthy school climate (Boykina, 2019; Dutta et al., 2021; Van Gils et al., 2024). Given this, understanding how interventions have adapted to emerging forms of bullying such as cyberbullying is crucial for developing effective responses.

In response to the evolving nature of bullying, UNESCO has recently introduced an inclusive and systemic definition that reconceptualizes bullying as a damaging social process embedded in institutional and societal norms, rather than as an individual or isolated behavioral event (O’Higgins Norman et al., 2024). This expanded definition highlights how power imbalances arise from broader social structures, peer-group norms, and school-level dynamics, rather than from differences in individual strength or personality alone. By emphasizing relational processes, social reinforcement mechanisms, and context-driven hierarchies, UNESCO calls for a ‘whole-education’ approach in which psychological interventions are understood not only as individual treatments but as strategies that must align with the social ecology of schools. This reconceptualization provides a foundation for analyzing contemporary interventions and understanding why systemic, multilayered approaches have gained relevance in recent decades.

The definition of bullying has evolved from an individualistic to a systemic and multidimensional perspective. While initially viewed as a problem stemming from individual traits like aggressiveness or lack of empathy (Kaliampos et al., 2022; Roland and Idsøe, 2001), later research has revealed the involvement of broader personal, family, school, and social dynamics (Febrianti et al., 2024; Swearer and Hymel, 2015). This shift has led psychological interventions to move from punitive actions toward comprehensive strategies that include victims, aggressors, teachers, and bystanders (Menesini and Salmivalli, 2017; Salmivalli, 2014). This expanded view sets the foundation for understanding how theory has guided intervention development.

The effectiveness of psychological interventions is rooted in the theoretical frameworks that guide their design. Cognitive-behavioral therapy (CBT) has shown effectiveness in increasing victims’ resilience and reducing aggressors’ violent behavior (Marande Perrin and Vernia Carrasco, 2022; Pinazo et al., 2020). Similarly, social–emotional learning programs have been introduced in schools to foster empathy and improve conflict resolution among students (Santamaría-Villar et al., 2021). However, their outcomes are uneven, highlighting the influence of institutional contexts and the challenges in teacher preparation that will be explored further.

Educational policies play a decisive role in the institutionalization and success of anti-bullying psychological interventions. In countries like Finland, mandatory implementation of intervention programs has contributed to a significant reduction in bullying prevalence (Salmivalli et al., 2011). In contrast, in contexts where school coexistence regulations are weak, interventions are inconsistent and yield limited results (Contreras Álvarez, 2013; Luzuriaga Torres and Ruiz Jara, 2021; Vega Báez, 2016). These disparities reveal the need to examine how policy environments enable or obstruct the effectiveness of school-based psychological strategies.

Institutional resistance remains a major barrier to the implementation of effective psychological interventions. In several regions, school bullying continues to be perceived as a natural part of development, which limits the adoption of structured prevention strategies (Mishna et al., 2006; Zhu, 2023). Moreover, the lack of administrative commitment and teacher overload often prevent the successful application of anti-bullying programs (Cunningham et al., 2016; Herkama et al., 2022). This persistent resistance reinforces the importance of aligning educational policies with the real conditions teachers face on the ground.

An interdisciplinary approach enriches our understanding of bullying and enhances the design of interventions. The Social-Ecological Theory has been widely applied to analyze how individual, family, school, and community factors influence bullying behaviors and intervention outcomes (Guo et al., 2021; Qiu, 2021). Additionally, social network analysis has revealed how peer group structures often reinforce exclusionary and aggressive patterns (Bass et al., 2022; Kornienko et al., 2020). These perspectives provide the analytical tools necessary to comprehend the broader systems that shape bullying dynamics and to identify effective points of intervention.

International experiences provide valuable lessons about the adaptability and scalability of psychological interventions. For example, the KiVa program in Finland focuses on altering the school climate and empowering students to respond to bullying (Clarkson et al., 2022; Cohane and Schneider, 2024), while Japan emphasizes early development of socio-emotional skills and school mediation practices (Hosokawa et al., 2023; Nakamichi et al., 2022). In contrast, Latin American efforts remain fragmented due to limited resources and inadequate teacher training (Dominguez-Rodriguez and De La Rosa-Gómez, 2022; González Moller et al., 2021). These case studies highlight the need for global perspectives that account for structural limitations and cultural diversity when designing interventions.

The rise of cyberbullying has disrupted traditional intervention models, demanding innovative responses from psychological approaches. Studies show that online bullying victims often experience more intense psychological distress due to the anonymity and permanence of digital aggression (Kim et al., 2023; Li et al., 2024). In response, new programs have integrated digital literacy and emotional coping strategies to equip students with tools to navigate virtual spaces safely (Buchan et al., 2024). These adaptations illustrate how psychological interventions must evolve in parallel with the changing nature of school bullying.

Artificial Intelligence (AI) presents both opportunities and ethical challenges in the fight against digital bullying. Machine learning algorithms have been implemented to detect aggressive behavior on educational platforms (Alqahtani and Ilyas, 2024; Herodotou et al., 2021). This duality calls for an integration of technological tools and human-centered psychological strategies to ensure responsible and effective intervention practices.

The latest technological trends have further complicated the landscape of school bullying, requiring rapid adaptation in intervention strategies. Advances such as deepfake technologies and viral offensive memes have added new layers to digital harassment, often escaping detection and regulation due to their novelty and speed (Mustak et al., 2023; Qazi and Hamid, 2023). Natural language processing tools, while useful for early detection, are being misused in parallel by aggressors for harm (Kara-Isitt et al., 2023). These developments emphasize the urgent need to strengthen digital literacy and embed critical media analysis in psychological interventions.

In light of the evolving nature of bullying, it is essential to examine how psychological interventions have changed and what strategies remain most effective today. This study aims to answer the following research question: *How have psychological interventions in school bullying evolved, and what are the most effective approaches currently?* To explore this question, five sub-questions will guide the analysis:

1. How have psychological interventions in bullying evolved in the last decade?
2. Which psychological approaches have demonstrated most effective in reducing bullying and supporting victims?
3. How do institutional norms and educational policies influence the implementation of psychological strategies?
4. What impact do these interventions have on students’ emotional well-being and academic achievement?
5. How can interventions be adapted to address new forms of bullying, including cyberbullying and AI-based aggression?

These guiding questions will support a comprehensive systematic review that explores emerging models, barriers to implementation, and context-sensitive solutions to bullying in schools.

## 2 Methodology

This study follows the guidelines of the PRISMA 2020 statement (Page et al., 2021) to conduct a systematic review of psychological interventions in school bullying. The review focused on empirical studies published between 2015 and 2024 and included only open-access articles.

### 2.1 Databases and search strategy

The search strategy used in this review employed the Boolean string (program OR intervention) AND bull AND (session). The term session was intentionally included to increase precision and retrieve studies reporting structured psychological sessions conducted by trained professionals. This focus aligns with the review’s objective of analyzing psychological interventions rather than general school-based actions or policy programs. However, we acknowledge that the use of session may have restricted the retrieval of studies that describe psychological interventions but do not explicitly use this term. Alternative strings such as (program OR intervention OR training OR workshop) AND bull AND (school OR education) could broaden the scope in future studies.

The literature search was conducted in three major academic databases: Scopus, Web of Science (WoS), and SciELO. To ensure full transparency and replicability, the search was restricted to Open Access (OA) publications. This decision was made to guarantee that all studies included in the review could be independently accessed, inspected, and reanalyzed by other researchers, which aligns with current open-science practices.

### 2.2 Inclusion and exclusion criteria

In this review, psychological interventions were defined as programs, techniques, or structured actions grounded in psychological theory and delivered with the primary aim of modifying emotional, cognitive, or behavioral processes related to bullying. These include, but are not limited to, cognitive-behavioral therapy (CBT), social–emotional skills training, counseling sessions, mindfulness-based approaches, and group-based emotional regulation activities. To maintain conceptual clarity, interventions whose main components were psychological—but delivered within school settings—were included even if teachers participated in the implementation.

School-wide programs, educational policies, or curricular reforms were not treated as psychological interventions. However, when such contextual elements substantially shaped the implementation or effectiveness of the psychological components (e.g., school climate policies supporting program adoption), they were analyzed only as contextual moderators, not as primary units of analysis. This distinction ensured that the synthesis remained focused on interventions grounded in psychological mechanisms, while still acknowledging institutional factors that influence their impact and sustainability.

Studies were included if they:

- focused on school populations (excluding higher education);
- involved psychological interventions aimed at addressing school bullying;
- reported empirical findings on the effectiveness or design of such interventions.

Exclusion criteria included:

- Duplicate records;
- Articles that addressed bullying but lacked a psychological component.

### 2.3 Selection process

The initial search yielded 197 records (Scopus = 111; WoS = 85; SciELO = 1). After removing 62 duplicates, 135 records were screened by title and abstract. Of these, 107 were excluded for not meeting the inclusion criteria. Full texts of the remaining 28 studies were assessed for eligibility, resulting in the exclusion of 7 reports (3 focused on higher education and 4 without psychological interventions). A total of 21 studies were included in the final review. This review intentionally limited the inclusion criteria to open-access articles to promote transparency, accessibility, and replicability, particularly for researchers, educators, and policymakers in low-resource settings. While this decision may have excluded some paywalled high-impact studies, it aligns with current open science practices and ensures that all cited evidence is freely available for verification and further research.

### 2.4 Quality appraisal

To assess the methodological rigor of the included studies, we conducted a structured quality appraisal using criteria adapted from the Mixed Methods Appraisal Tool (MMAT, 2018) and the CASP checklists for qualitative and quantitative evidence. Rather than applying a numerical scoring system—which is discouraged by MMAT developers—the appraisal focused on evaluating studies across four core domains:

1. Clarity and coherence of the research question;
2. Adequacy of the methodological design (including sampling, data collection procedures, and intervention description);
3. Transparency and robustness of the outcome measures; and
4. Alignment between methods, analyses, and reported findings.

**Figure 1:**
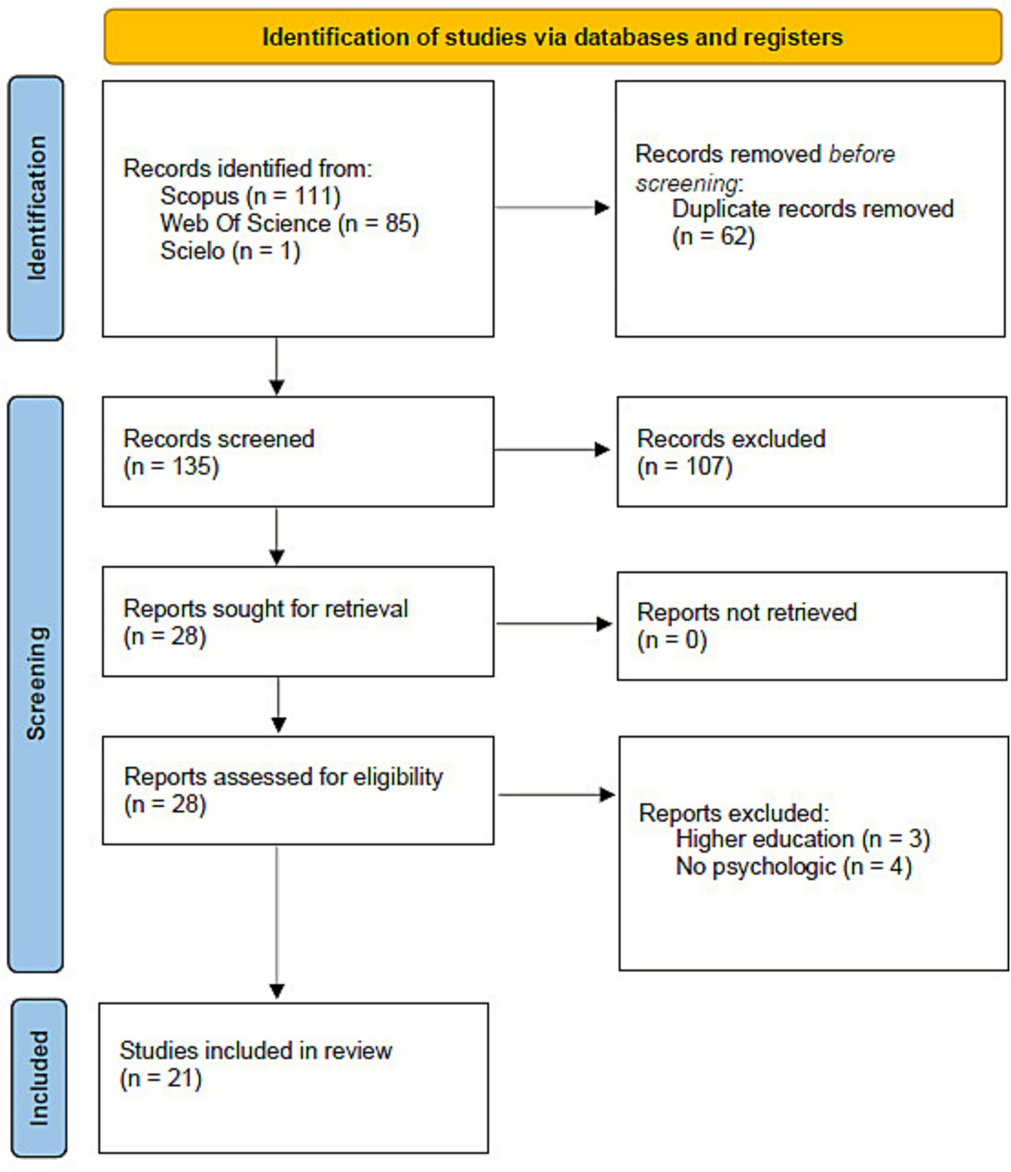
PRISMA 2020 flow diagram for study selection.

Each study was reviewed independently by two researchers, and studies presenting unclear intervention protocols, missing information about sample characteristics, lack of follow-up measurements, or insufficient description of analytic procedures were flagged as presenting higher risk of bias. Although all studies were retained—consistent with PRISMA guidelines—the quality appraisal informed the interpretation of the evidence synthesis, particularly when contrasting findings across diverse designs and geographical contexts. The appraisal results are summarized in the final table of included studies, where methodological limitations are explicitly noted to enhance transparency and contextualize the strength of the conclusions. The study selection process is illustrated in Figure 1, following the PRISMA flow diagram.

## 3 Results

To ensure transparency and replicability, the complete dataset used for this systematic review—including the list of included studies, extracted variables, and exclusion justifications—has been made publicly available through the Zenodo open-access repository (https://zenodo.org/records/15091482). In addition, a complementary summary table is included at the end of this manuscript (Table 1). The first outlines the key characteristics of each included study (e.g., author, year, country, intervention type, sample size), while the second presents a comparative overview of study designs, theoretical frameworks, and outcome categories. The reviewed studies represent a broad geographical distribution—including Latin America, Europe, Asia, and North America—which strengthens the external validity of the findings and ensures that conclusions are not regionally biased.

**Table 1:** Papers included.

Authors	Title	Year	Country	Methodology	Outcome measured	Main findings	Population
Jacobs N. C.; Völlink T.; Dehue F.; Lechner L.	Online Pestkoppenstoppen: Systematic and theory-based development of a web-based tailored intervention for adolescent cyberbully victims to combat and prevent cyberbullying	2014	Netherlands	Intervention Mapping protocol with six steps Pretesting and implementation planning Randomized controlled trial (RCT) with experimental, general information, and waiting list control groups - Self-reported questionnaires for outcome measurement	Frequency of (cyber)bullying, psychological wellbeing, problem behavior, school performance, and truancy	The paper describes the systematic development of the Online Pestkoppenstoppen intervention, a web-based tailored program for adolescent cyberbully victims. The intervention aims to increase psychological well-being and decrease victimization, problem behavior, school problems, and truancy.	Adolescents aged 12 to 15 years old from Continued Secondary Vocational Education
Nakamura R.; Koshikawa F.	Evaluating a psycho-educational program for the prevention of bullying in junior high school	2014	Japan	Qualitative design The study involved a psychoeducational program aimed at preventing bullying among junior high school students. The program consisted of psychological education and role-playing. Psychological education focused on the unacceptability of bullying and creating a bullying-free environment.	Self-efficacy regarding intervention actions against bullying, normative consciousness against bullying, tendency to bully	The bullying prevention program improved self-efficacy for stopping bullying and anti-bullying norms, and reduced the tendency to bully. The program significantly reduced the tendency to commit bullying. The program was effective in strengthening anti-bullying norms, particularly in students with initially low anti-bullying norm consciousness.	Junior high school students, likely around 13 to 14 years old
Garaigordobil M.; Martínez-Valderrey V.	Effect of Cyberprogram 2.0 on reducing victimization and improving social competence in adolescence; [Efecto del Cyberprogram 2.0 sobre la reducción de la victimización y la mejora de la competencia social en la adolescencia]	2014	Spain	Implementation of Cyberprogram 2.0 with 19 one-hour sessions. Four main objectives: identify and conceptualize bullying, analyze consequences, develop coping strategies, and promote positive social behaviors. Quasi-experimental design with pretest-posttest control group. Sample of 176 adolescents aged 13 to 15. Evaluation using two psychometrically validated instruments. Data analysis: descriptive statistics, ANOVAs for pretest, ANCOVAs for posttest.	Victimización por bullying presencial (AVE) Conductas sociales positivas (AECS): conformidad social, ayuda-colaboración, seguridad-firmeza, liderazgo prosocial Conductas sociales negativas (AECS): agresividad-terquedad, dominancia, apatía-retraimiento, ansiedad social	The Cyberprogram 2.0 significantly reduced bullying victimization and increased positive social behaviors such as social conformity, help-cooperation, security-firmness, and prosocial leadership. Women showed a greater decrease in negative social behaviors like aggression, dominance, and apathy compared to men.	Adolescents aged 13 to 15 years old, in 3rd and 4th grade of Compulsory Secondary Education
Tawalbeh A. H.; Abueita J. D.; Mahasneh A.; Shammout N.	Effectiveness of a counseling program to improve self-concept and achievement in bully-victims	2015	Jordania	Participants: Sixth-grade pupils (ages 12–13) who were victims of bullying. Intervention: 10 sessions of group counseling based on CBT (PRACTICE model). Groups: Experimental group received counseling; control group did not. Assessments: Pre- and post-tests using Self Concept Scale and Bully Behavior Scale. Statistical Analysis: T-tests to compare experimental and control groups.	Self-concept, bullying victim behavior, and academic achievement	The study found significant statistical differences between the experimental and control groups in self-concept, bullying victim behavior, and achievement after the counseling program. The experimental group showed improvement in self-concept and achievement, and a decrease in bullying behavior compared with the control group. The study concluded that the counseling program had a positive effect on these areas.	Male sixth-grade pupils, aged 12 to 13 years old
Sosin L. S.; Rockinson-Szapkiw A. J.	Creative Exposure Intervention as Part of Clinical Treatment for Adolescents Exposed to Bullying and Experiencing Posttraumatic Stress Disorder Symptoms	2016	USA	Qualitative design The Creative Exposure Intervention combines cognitive-behavioral therapy (CBT), mindfulness techniques, and art therapy techniques. The intervention is structured into four steps: emotion regulation and safe place phase, bullying-related imagery exposure phase, solution imagery phase, and application phase.	Reduction in Subjective Units of Disturbance (SUDS) and Belief Levels (BL) (Scale: 1–10)	The Creative Exposure Intervention is introduced as a treatment approach for adolescents with PTSD symptoms due to bullying, combining cognitive-behavioral therapy, mindfulness, and art therapy. The intervention aims to re-establish attachment security, discuss bullying experiences, reframe negative narratives, and plan for future safety and identity development.	Adolescents
Garaigordobil M.; Martínez-Valderrey V.	Impact of cyberprogram 2.0 on different types of school violence and aggressiveness	2016	Spain	Repeated measures pre-posttest design with a control group 19 one-hour sessions for the intervention Program objectives: identify and conceptualize bullying/cyberbullying, analyze consequences, develop coping strategies, achieve transversal goals Administered by a trained adult in a group setting	School violence: Teachers’ violence toward students Students’ physical violence Students’ verbal violence Social exclusion Violence through ICT Premeditated aggressiveness Impulsive aggressiveness	The Cyberprogram 2.0 intervention significantly reduced various types of school violence, including teachers’ violence toward students, students’ physical and verbal violence, social exclusion, and violence through ICT.	Teenagers aged 13 to 15 years old, specifically Secondary Education students in grade 8.
Rajabi M.; Bakhshani N.-M.; Saravani M. R.; Khanjani S.; Bagian M. J.	Effectiveness of cognitive-behavioral group therapy on coping strategies and in reducing anxiety, depression, and physical complaints in student victims of bullying	2017	Iran	Quasi-experimental study with pretest-posttest control group design Data collection using Olweus Bully/Victim Questionnaire, Achenbach’s Youth Self-Report (YSR), and Billings and Mouse’s Coping Strategies Scale Participants randomly assigned to experimental or control group Experimental group received cognitive-behavioral group therapy over 12 sessions	Anxiety, depression, physical complaints, emotion-focused coping strategies, problem-focused coping strategies	Cognitive-behavioral group therapy significantly reduced anxiety, depression, and physical complaints in student victims of bullying. The therapy improved coping strategies by increasing problem-focused strategies and decreasing emotion-focused strategies.	Male students in the city of Zahedan studying in the 2014–2015 academic year
Vanega-Romero S.; Sosa-Correa M.; Castillo-Ayuso R.	Bullying, anger and depression in Mexican adolescents: A preliminary study of the effectiveness of an intervention.; [Acoso escolar, ira y depresión en adolescentes mexicanos: Un estudio preliminar de la eficacia de una intervención]	2018	Mexico	Mixed-methods design Stratified cluster sampling for participant selection Use of scales: Escala de violencia entre pares, Staxi-NA, CDI Semi-structured interviews and K-SADS-PL for qualitative data	Victimización (victimization) Rasgos depresivos (depressive symptoms) Ira rasgo (trait anger) Ira expresión (anger expression) Ira control (anger control)	The intervention effectively reduced victimization and improved anger control in both victims and aggressors. The intervention successfully reduced symptoms associated with school bullying and improved anger management.	Secondary school students aged 12 to 16 years old
Garaigordobil M.; Martínez-Valderrey V.	Technological resources to prevent cyberbullying during adolescence: The Cyberprogram 2.0 program and the cooperative Cybereduca 2.0 Videogame	2018	Spain	Quasi-experimental design with repeated pretest-posttest measures and control groups. Experimental group received intervention with weekly 1-h sessions over the school year. Control group followed usual school activities. Eight assessment instruments administered during pretest and posttest stages.	Reduction in bullying and cyberbullying behaviors Improved perception of school violence Decrease in aggressiveness Increase in positive social behaviors	The Cyberprogram 2.0 and Cooperative Cybereduca 2.0 intervention significantly reduced face-to-face and cyberbullying behaviors in adolescents. The intervention improved perception of school violence, decreased aggressiveness, and increased positive social behaviors, self-esteem, and empathy.	Adolescents aged 13 to 15 years old
Schoeps K.; Villanueva L.; Prado-Gascó V. J.; Montoya-Castilla I.	Development of emotional skills in adolescents to prevent cyberbullying and improve subjective well-being	2018	Spain	Quasi-experimental design with randomized classroom assignment Intervention based on Mayer and Salovey’s emotional intelligence model Eleven sessions over three months Path analysis using Mplus software	Reduction of victimization and assault via mobile phones and the Internet Enhancement of emotional perception and regulation skills Increase in life satisfaction	The intervention program reduced cyberbullying behaviors, specifically victimization and assault via mobile phones and the Internet.	Adolescents aged 12 to 15 years, specifically 7th and 8th grade students of secondary school.
Silva J. L. D.; Oliveira W. A.; Carlos D. M.; Lizzi E. A. D. S.; Rosário R.; Silva M. A. I.	Intervention in social skills and bullying	2018	Brasil	Quasi-experimental study design Cognitive-behavioral intervention based on social skills Eight weekly sessions addressing politeness, friendships, self-control, emotional expressiveness, empathy, assertiveness, and interpersonal problem-solving Pre-test, post-test, and follow-up evaluations using EVAP and SMHSC scales Data analysis using Poisson regression with random effect Conducted in six public schools with a follow-up evaluation 1 year later	Bullying victimization, difficulty in social skills	The intervention significantly reduced the difficulty in social skills for bullying victims, with this improvement persisting over time. There was a significant reduction in total victimization in both the intervention and control groups. The intervention group showed significant reductions in verbal and relational victimization, while the control group showed significant reductions in physical victimization.	6th grade students, approximately 11 years old
Yan H.; Chen J.; Huang J.	School bullying among left-behind children: The efficacy of art therapy on reducing bullying victimization	2019	China	Sampled 603 children from 6 rural schools. Used questionnaires to assess psychological and school behavior status. Conducted interventions over 6 sessions.	Bullying victimization; school life satisfaction; self-esteem	Left-behind children experienced more bullying victimization than non-left-behind children. Left-behind boys were more likely to be bullied than left-behind girls. Art therapy significantly reduced bullying victimization among left-behind children.	Fifth-grade students in rural areas, approximately 10 to 12 years old
Goodwin, J; Bradley, SK; Donohoe, P; Queen, K; O’Shea, M; Horgan, A	Bullying in Schools: An Evaluation of the Use of Drama in Bullring Prevention	2019	Ireland	Qualitative descriptive research Focus group interviews with students from six schools 50 students participated in focus groups Investigator triangulation for enhanced rigor	Student experience and engagement with the Bullying Prevention Session (BPS) Implementation of recommendations from BPS workshops in the school post-intervention	The Bullying Prevention Session (BPS) improved students’ understanding of bullying complexities, including perspectives of both bullies and bystanders. Students were dissatisfied with the lack of implementation of their suggested bullying reduction strategies by the schools.	Teenagers aged 12 to 15 years old
Aliyar Najafabadi R.; Meshkati Z.; Badami R.	The Effectiveness of Assertiveness Training on Bullying, Competitive State Anxiety and Performance Under Pressure in Futsal Players	2020	Iran	Study design: Controlled quasi-experimental field study with Pre-test-Post-test design. Participant selection: Targeted sampling, randomly divided into experimental and control groups. Intervention: Experimental group received 8 assertiveness training sessions. Data collection: Gambrill-Richey assertiveness inventory, University of Illinois bully scale, competitive state anxiety inventory-2, athletic performance questionnaire.	Bullying, competitive state anxiety, performance under pressure	Assertiveness training significantly reduced bullying in adolescent male futsal players. Assertiveness training significantly reduced competitive state anxiety in adolescent male futsal players. Assertiveness training significantly improved performance under pressure in adolescent male futsal players.	adolescent male futsal players aged 15–17 years old in Isfahan City, Iran
Ortega-Barón J.; González-Cabrera J.; Machimbarrena J. M.; Montiel I.	Safety. Net: A pilot study on a multi-risk internet prevention program	2021	Spain	Study design: Pre/post-test repeated measures design with a control group and an intervention group. Sample: 165 adolescents aged 11 to 14 years old (120 intervention, 45 control). Sampling method: Nonprobability convenience sampling. Data collection: Pre-test in December 2019, post-test in May–June 2020 during COVID-19 lockdown.	Online grooming, problematic Internet use, Internet gaming disorder, and nomophobia	The Safety.net program demonstrated improvements in preventing online grooming, problematic Internet use, Internet gaming disorder, and nomophobia compared to the control group. Significant interaction effects were found for problematic Internet use, Internet gaming disorder, and nomophobia, indicating the program’s effectiveness.	Adolescents aged 11 to 14 years old
Montero-Carretero C.; Roldan A.; Zandonai T.; Cervelló E.	A-judo: An innovative intervention programme to prevent bullying based on self-determination theory—a pilot study	2021	Spain	Experimental and control groups were used. Physical education teachers received 20 h of training. The A-Judo Programme consisted of 10 sessions over 5 weeks. Methodologies included student choice, problem-solving, achievable goals, positive reinforcement, technical feedback, and cooperative work. The programme aimed to prevent bullying and promote moral and social development through judo. A pre-post-test quasi-experimental design was used to assess effectiveness. Data collection involved student questionnaires before and after the intervention.	Bullying, Victimization	The A-Judo Programme significantly improved autonomy support, basic psychological needs satisfaction, intrinsic motivation, moral identity, and tolerance-respect among students. The intervention group showed lower levels of control style, external motivation, and bullying compared to the control group. The program was effective in reducing bullying but had limitations in addressing victimization behavior.	Children around the age of 11 (average age 11.13 years, SD = ±0.52 years)
Jueajinda S.; Stiramon O.; Ekpanyaskul C.	Social intelligence counseling intervention to reduce bullying behaviors among thai lower secondary school students: A mixed-method study	2021	Thailand	Mixed-method design Phase 1: Qualitative research using interviews with key informants to develop the counseling program Phase 2: Randomized controlled trial with a wait-list control to evaluate the program’s effectiveness Purposive sampling of lower secondary school students with bullying behaviors	Social Intelligence Scale (SIS) scores, Bullying-Behavior Scale (BBS) scores	The social intelligence counseling program significantly enhanced social intelligence scores in the experimental group compared to the control group. Promoting social awareness was identified as a key factor in reducing bullying behaviors among students.	Lower secondary school students in Bangkok, Thailand, with repeated bullying behaviors
Peng Z.; Li L.; Su X.; Lu Y.	A pilot intervention study on bullying prevention among junior high school students in Shantou, China	2022	China	Four-session educational intervention based on the knowledge-attitude-practice model. Intervention methods: bullying-themed class meetings, distribution of educational leaflets, playing anti-bullying videos. Pre-post intervention research design. Data collection: questionnaires before and after intervention. Statistical analysis: chi-square tests, t-tests.	Awareness of bullying, Acceptance of anti-bullying education, Incidence of cyber victimization, Incidence of social victimization.	The intervention significantly improved awareness of bullying among both male and female students. There was a significant reduction in the incidence of cyber victimization among both male and female students. The study observed reductions in social, verbal, peer, and social bullying among male students.	Junior high school students, approximately 12.8 to 12.9 years old
Williams C.; Griffin K. W.; Botvin C. M.; Sousa S.; Botvin G. J.	Effectiveness of Digital Health Tools to Prevent Bullying among Middle School Students	2023	USA	Cluster-randomized comparison group design with 14 middle schools. Intervention included classroom sessions and digital materials for students, parents, and school staff. Online pre- and post-test surveys to assess bullying-related behavior, knowledge, and life skills. Statistical analyses: GLM and multilevel models controlling for pre-test scores, race/ethnicity, and gender. One-tailed significance tests for hypothesis testing.	Frequency of bullying perpetration Frequency of bullying victimization Overall Skills Knowledge Specific skills knowledge (Bullying Prevention, Self-Image, Decision Making, Coping with Anxiety, Coping with Anger, Assertiveness)	Students in the intervention group reported significantly less bullying and cyberbullying perpetration compared to those in the comparison group. The intervention resulted in increased life skills knowledge among the students in the intervention group.	Middle school students aged 11–14 years old, primarily in grades 6–8.
Lee M.-B.; Yeom Y. O.; Kim M. S.; Lee Y.; Kim K. M.; Kim D. H.; Lee C. M.; Lim M. H.	Effects of school sandplay group therapy on children victims of cyberbullying	2023	Korea	Study design: Parallel-group non-randomized controlled trial Intervention: 10 sessions of sandplay group therapy (SSGT) for 40 min per session Assessment tools: Children Depression Inventory, Suicidal Ideation Questionnaire-Junior, Rosenberg Self-Esteem Scale Data analysis: Multivariate analysis of variance Program structure: Based on communicative sandplay and Kalff stages of ego-development	Depression (measured by Children Depression Inventory) Suicidal ideation (measured by Suicidal Ideation Questionnaire-Junior) Self-esteem (measured by Rosenberg Self-Esteem Scale)	The SSGT group showed a significant decrease in depression and suicidal ideation compared to the control group after sandplay group therapy. SSGT significantly increased self-esteem in children who were victims of cyberbullying.	Elementary school students, 5th and 6th grade, aged 11 to 12 years, residing in Cheonan City, Korea
Badger J. R.; Rovira A.; Freeman D.; Bowes L.	Developing a virtual reality environment for educational and therapeutic application to investigate psychological reactivity to bullying	2023	United Kingdpm	Convenience sample of 67 female adolescents. Participants assigned to neutral or hostile VR scenarios based on anxiety, depression, paranoia, and previous bullying. Use of Oculus Rift CV1 for VR experience. Pre- and post-VR questionnaires for negative affect and distress. Linear regression models for data analysis.	Self-reported distress, negative affect	Participants who experienced the hostile VR scenario reported significantly greater negative affect compared to those in the neutral scenario. The study supports the use of VR for recreating hostile social situations and assessing real-time psychological reactivity in bullying contexts.	Female adolescents aged 11–15 years old from U. K. secondary schools

### 3.1 How have psychological interventions for bullying evolved over the last decade?

In the last decade, psychological interventions targeting school bullying have undergone a significant theoretical and methodological transformation. Initially rooted in individual-behavioral approaches, these interventions have evolved toward systemic, multidimensional strategies that integrate emotional, social, and cognitive-behavioral components. This evolution is not only theoretical but also contextual, as variations in implementation reflect the diversity of educational systems and cultural environments.

During the early 2010s, psychological interventions were primarily behaviorist, focusing on anger control and emotional skills through CBT-based programs such as those implemented in Yucatán, Mexico, which showed reductions in depressive symptoms and aggression but lacked school-wide integration (Vanega-Romero et al., 2018). As the decade progressed, interventions adopted broader theoretical models like the social-ecological framework, emphasizing peer relationships, empathy, and cooperative learning to reduce victimization and promote prosocial behavior (Garaigordobil and Martínez-Valderrey, 2014). Later developments introduced symptom-specific targeting, with group interventions for adolescents with high anger or depressive traits yielding positive emotional outcomes (Vanega-Romero et al., 2018). The growing prevalence of cyberbullying further pushed innovations, leading to hybrid interventions combining digital literacy and emotional regulation, though systematic reviews still note a shortage of validated programs specifically designed for online aggression (Garaigordobil and Martínez-Valderrey, 2014).

Regionally, the evolution of interventions has reflected both innovation and disparity. In Europe, especially in Spain and Finland, structured, evidence-based programs like KiVa have been institutionalized with strong policy backing and school-level integration (Ortega-Barón et al., 2021). These models are informed by rigorous experimental designs and long-term evaluation. In contrast, interventions in Latin America often stem from academic or community initiatives with limited scalability due to structural barriers such as lack of funding or institutional resistance (Vanega-Romero et al., 2018). For instance, the Yucatán-based program demonstrated promising results but lacked a control group and follow-up assessments, limiting its generalizability.

**Figure 2:**
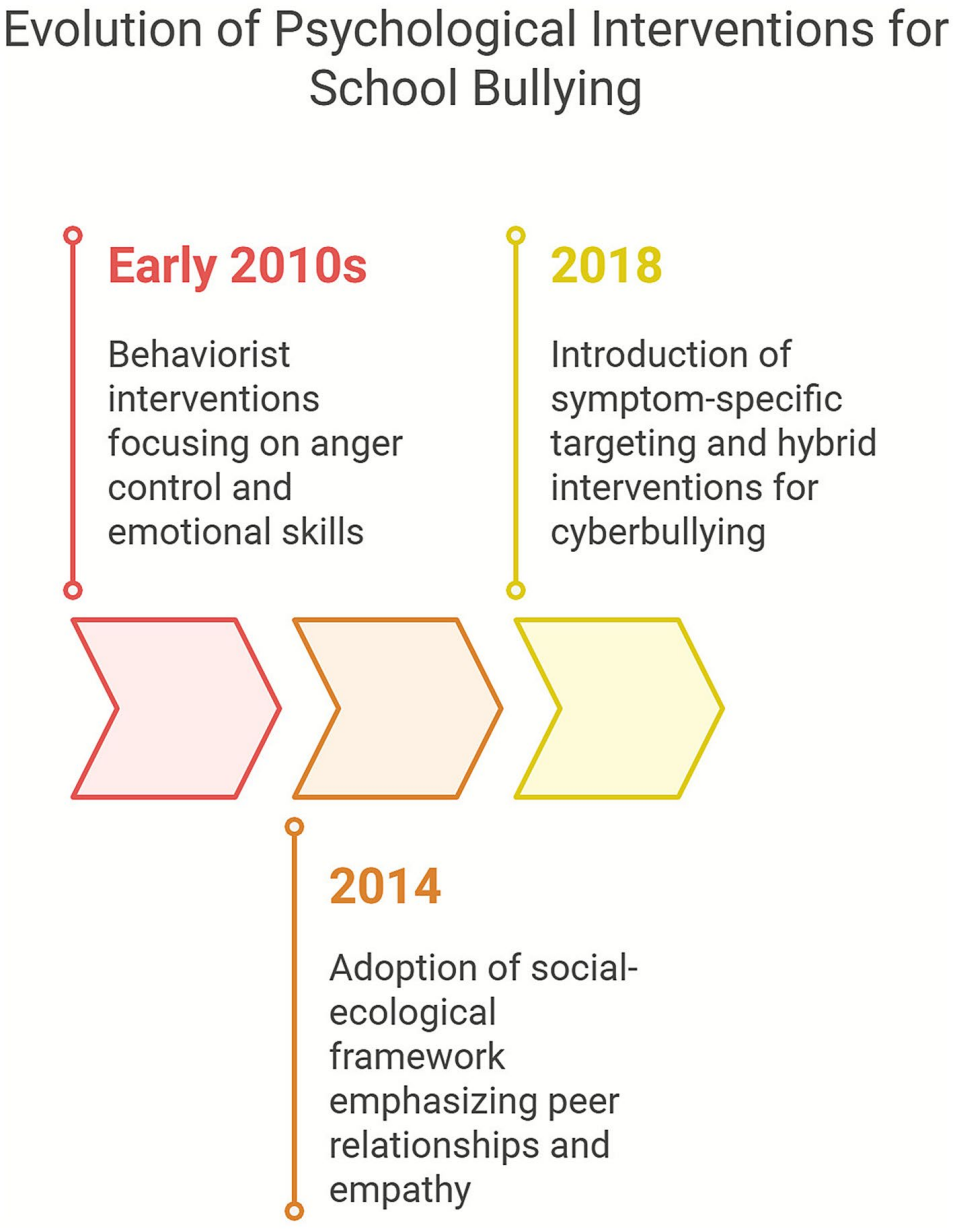
Evolution of psychological interventions for school bullying.

In conclusion, over the past ten years, psychological interventions for school bullying have evolved from isolated cognitive-behavioral strategies toward comprehensive, systemic approaches grounded in social-ecological and developmental frameworks. They now increasingly integrate emotional literacy, digital education, and personalized therapeutic techniques. However, their evolution remains uneven across regions, underscoring the importance of contextual adaptation and institutional support to ensure effectiveness and sustainability (Figure 2).

### 3.2 Which psychological approaches have demonstrated most effective in reducing bullying and supporting victims?

Cognitive-behavioral therapy (CBT) has been one of the most frequently applied and empirically supported approaches for reducing bullying behaviors. Group interventions focusing on managing anger, depression, and anxiety have shown significant reductions in aggressive behavior and emotional symptoms among adolescents involved in school bullying (Lee et al., 2023; Ortega-Barón et al., 2021; Rajabi et al., 2016; Vanega-Romero et al., 2018). For instance, programs implemented in Mexican and Spanish contexts demonstrated clinical improvements after structured CBT sessions incorporating techniques such as role-play, relaxation, and cognitive restructuring (Ortega-Barón et al., 2021; Vanega-Romero et al., 2018). Moreover, this approach proved particularly effective for students with more intense emotional profiles, such as high trait anger or moderate depressive symptoms.

Social–emotional learning (SEL) has also shown strong effectiveness, particularly in reducing victimization and increasing prosocial behaviors. Interventions including empathy training, emotional regulation, and conflict resolution led to improvements in school climate and students’ perception of safety (Garaigordobil and Martínez-Valderrey, 2016, 2018, 2018). Programs like *Cyberprogram 2.0* integrated SEL components within a solid cognitive-behavioral framework, producing reductions in direct aggression and peer rejection. However, some studies reported gender-based differences in outcomes, with girls often showing greater improvement in prosocial behaviors.

CBT and SEL interventions have shown effectiveness under specific conditions, rather than as universally superior approaches, and their outcomes depend heavily on contextual moderators such as teacher training, fidelity of implementation, cultural norms, and availability of institutional resources.

**Figure 3:**
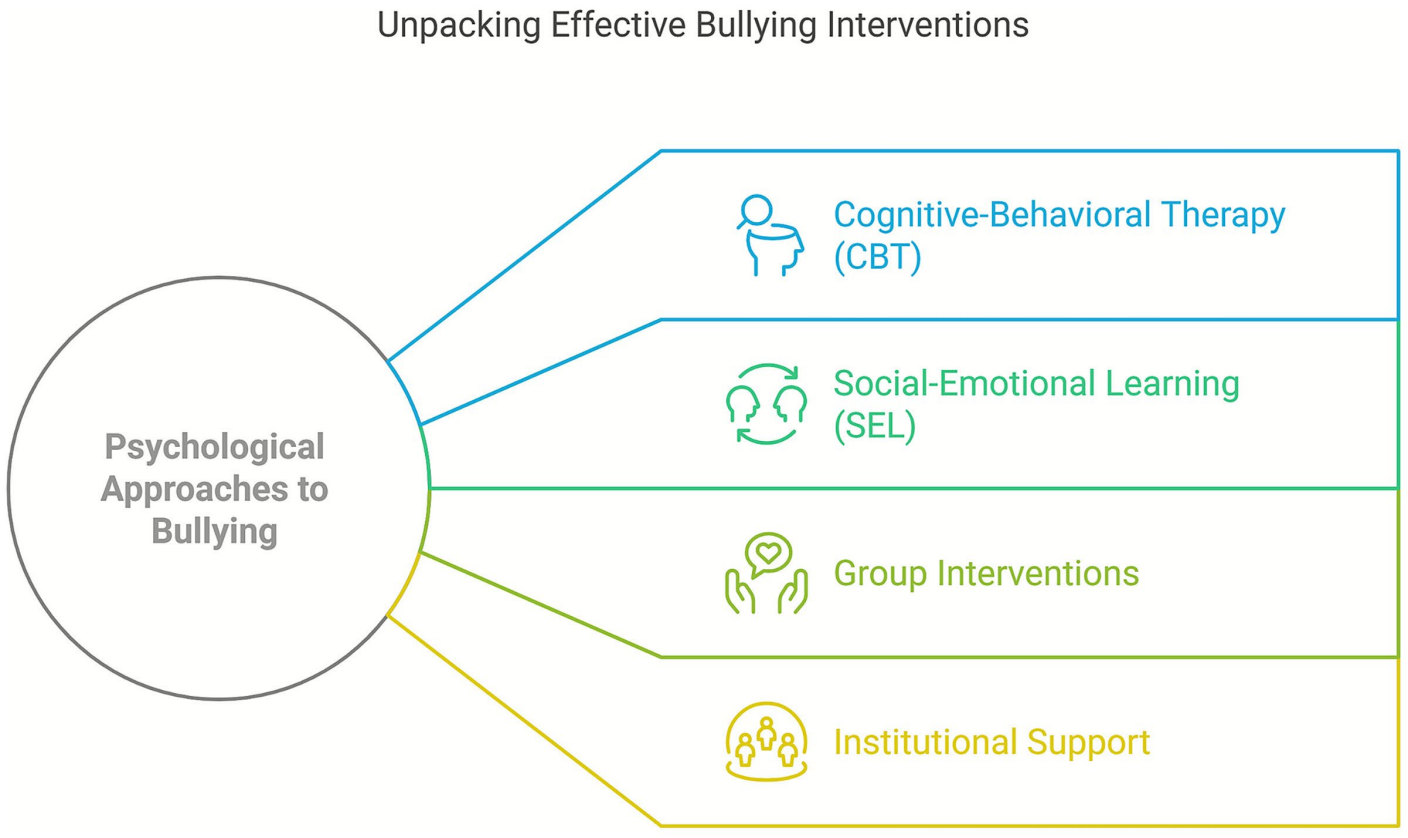
Effective bullying interventions.

Although both approaches have demonstrated effectiveness, limitations were noted when they were not implemented in conjunction with broader school-wide strategies. Isolated interventions without active involvement from teachers or without parallel work with bystanders tended to show reduced or short-lived effects (Ortega-Barón et al., 2021; Tawalbeh et al., 2015; Vanega-Romero et al., 2018; Williams et al., 2023). These findings suggest that while CBT and SEL offer robust theoretical foundations, their impact is significantly enhanced when applied comprehensively, with institutional support and strategies that engage the entire educational community (Figure 3).

### 3.3 How do institutional norms and educational policies influence the implementation of psychological strategies?

The presence or absence of solid normative frameworks directly influences the viability and sustainability of psychological strategies against bullying. In Latin American countries such as Mexico, there is a high prevalence of school bullying, partly explained by the lack of clear institutional protocols and limited operationalization of public policies regarding school coexistence (Vanega-Romero et al., 2018). Reports cited in the OECD studies reflect Mexico’s elevated levels of victimization, despite official diagnoses acknowledging the problem. This disconnect between recognition and action suggests that without a regulatory structure requiring interventions and aligning them with the educational system, isolated psychological efforts tend to lose momentum and continuity.

Internal school regulations can also serve as either barriers or facilitators for implementing psychological strategies. Some studies revealed that even when intervention programs yielded positive outcomes, their sustainability was limited due to a lack of integration into school rules or the absence of coordination among teachers and support staff (Silva et al., 2018; Yan et al., 2019). These programs often depend on the initiative of individual psychologists or researchers and lack formal institutional support, which prevents their long-term consolidation. Therefore, incorporating these strategies into school coexistence plans, internal manuals, or inclusive policies is essential to ensure their lasting effectiveness.

**Figure 4:**
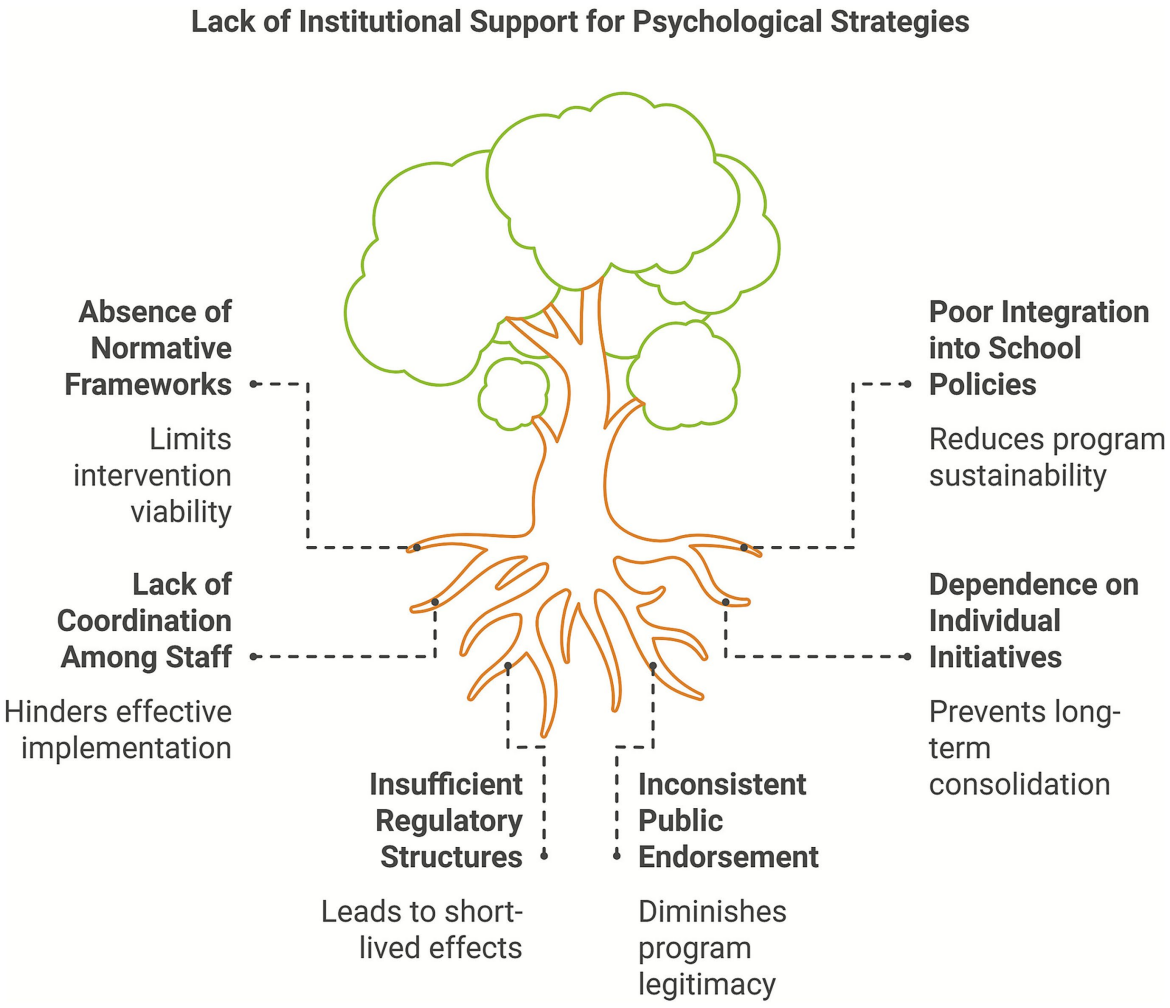
Lack of institutional support for psychological strategies.

International comparisons highlight clear contrasts between systems with strong regulatory frameworks and those without defined policies. In Spain, for instance, educational policies have supported programs such as SAVE and Cyberprogram 2.0, facilitating their institutional validation and integration into the school curriculum (Garaigordobil and Martínez-Valderrey, 2018). These programs have benefited from public endorsement and are part of broader strategies for school violence prevention. Likewise, international studies referenced in the same work recognize Finland as a benchmark due to the national implementation of the KiVa program and its articulation with educational coexistence policies (Garaigordobil and Martínez-Valderrey, 2014, 2016). These findings reinforce that psychological strategies cannot be effectively deployed without alignment with educational norms and policies that grant them legitimacy, resources, and long-term sustainability (Figure 4).

### 3.4 What impact do these interventions have on students’ emotional well-being and academic achievement?

**Figure 5:**
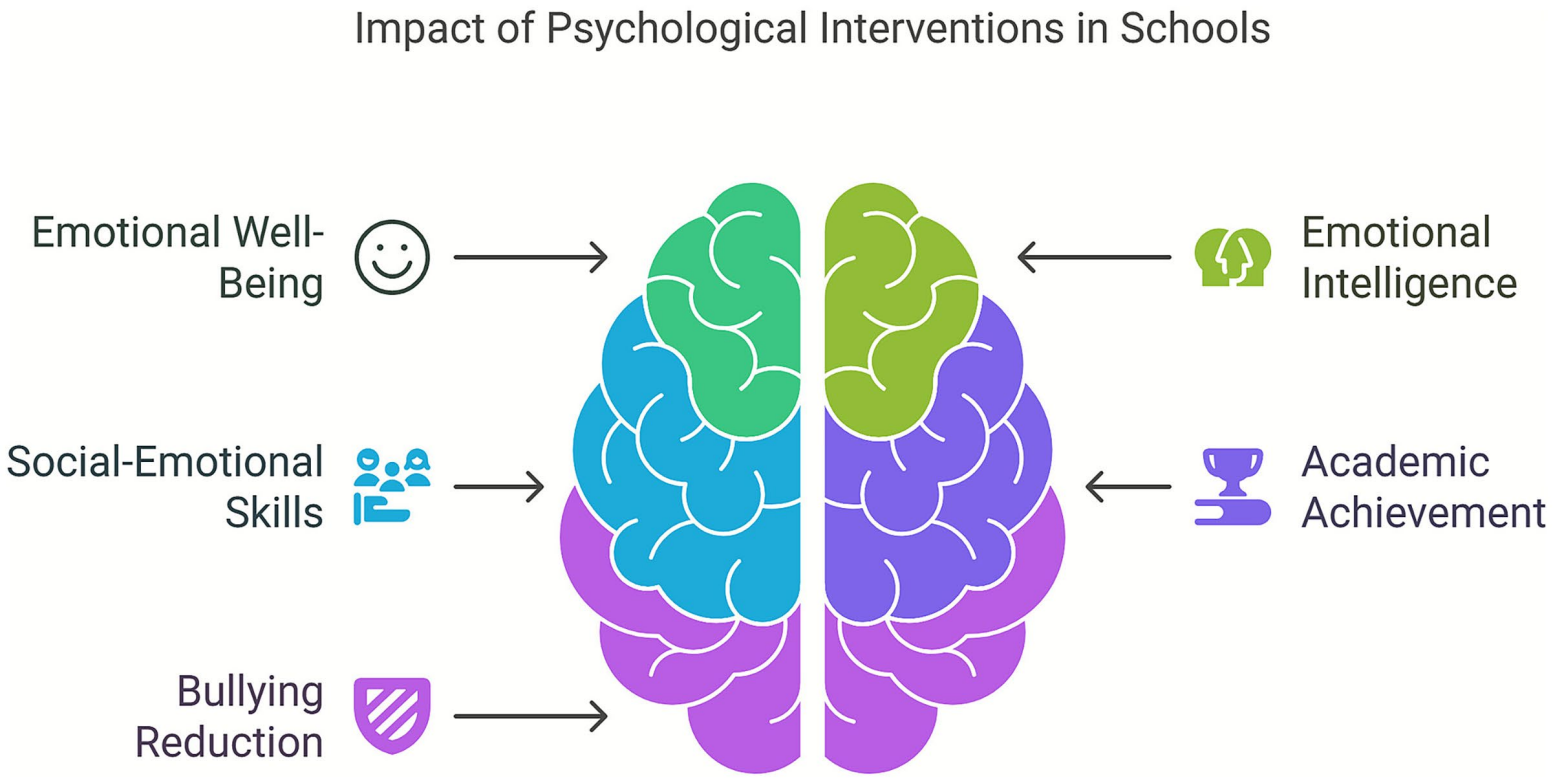
Impact of psychological interventions in schools.

Psychological interventions have shown consistent positive effects on students’ emotional well-being, particularly in reducing internalizing symptoms such as anxiety, anger, and depression. For instance, Vanega-Romero et al. (2018) reported significant decreases in depressive symptoms and internalized anger among both victims and aggressors following a CBT-based intervention implemented in Mexican secondary schools. Similarly, Garaigordobil and Martínez-Valderrey (2016) found that participants in the Cyberprogram 2.0 showed improvements in emotional regulation and empathy, as well as reduced victimization and aggressiveness. On the academic side, Tawalbeh et al. (2015) documented a significant increase in students’ academic performance and self-concept after a school-based counseling program in Jordan. While not all interventions reported academic outcomes explicitly, several studies noted indirect improvements in school engagement and social integration, which are known to correlate with academic success (Ortega-Barón et al., 2021; Sosin and Rockinson-Szapkiw, 2016). Overall, the evidence suggests that psychological interventions not only promote emotional resilience but can also contribute to improved academic outcomes when implemented in a supportive school context (Figure 5).

### 3.5 How can interventions be adapted to address new forms of bullying, including cyberbullying and AI-based aggression?

The discussion of deepfakes, algorithmic exclusion, and offensive memes corresponds to emerging risks identified in the broader literature, which were not empirically examined in the studies included in this review. These trends are therefore presented as a future agenda, rather than as part of the current evidence base.

The digitalization of school interactions has led to the emergence of new bullying modalities, including cyberbullying and AI-mediated aggression, which challenge the design and scope of traditional psychological interventions. Empirical evidence from the reviewed literature indicates that digital aggression differs from face-to-face bullying in key aspects such as persistence, anonymity, and reach, often intensifying the psychological harm and reducing schools’ ability to respond effectively (Garaigordobil and Martínez-Valderrey, 2014; Vanega-Romero et al., 2018). For instance, NLP-based algorithms have begun to support the early detection of online bullying by analyzing digital messages and flagging harmful language (Badger et al., 2023), allowing for more timely psychological support. However, these tools are still limited in reach and ethical safeguards, and often remain outside school-wide prevention frameworks.

In contrast, emerging risks such as the use of deepfakes, offensive memes, or algorithmic exclusion in digital platforms represent novel forms of bullying for which no validated psychological interventions currently exist. These expressions are harder to detect, evolve rapidly, and are often masked under humor or virality, making them more difficult to regulate within educational contexts (Kara-Isitt et al., 2023; Vanega-Romero et al., 2018). The reviewed studies emphasize the importance of preparing students and staff through digital literacy training, emotional resilience development in virtual environments, and critical media consumption as part of broader prevention efforts (Garaigordobil and Martínez-Valderrey, 2014; Vanega-Romero et al., 2018).

Beyond the empirical evidence found in the reviewed articles, the broader literature identifies emerging digital threats—including deepfake-based harassment, algorithmic exclusion, and the viral spread of offensive memes—that challenge traditional psychological interventions. While these phenomena were not directly examined in the included studies, they represent critical future risks that require the development of prevention strategies integrating digital literacy, media analysis, and cross-disciplinary collaboration.

**Figure 6:**
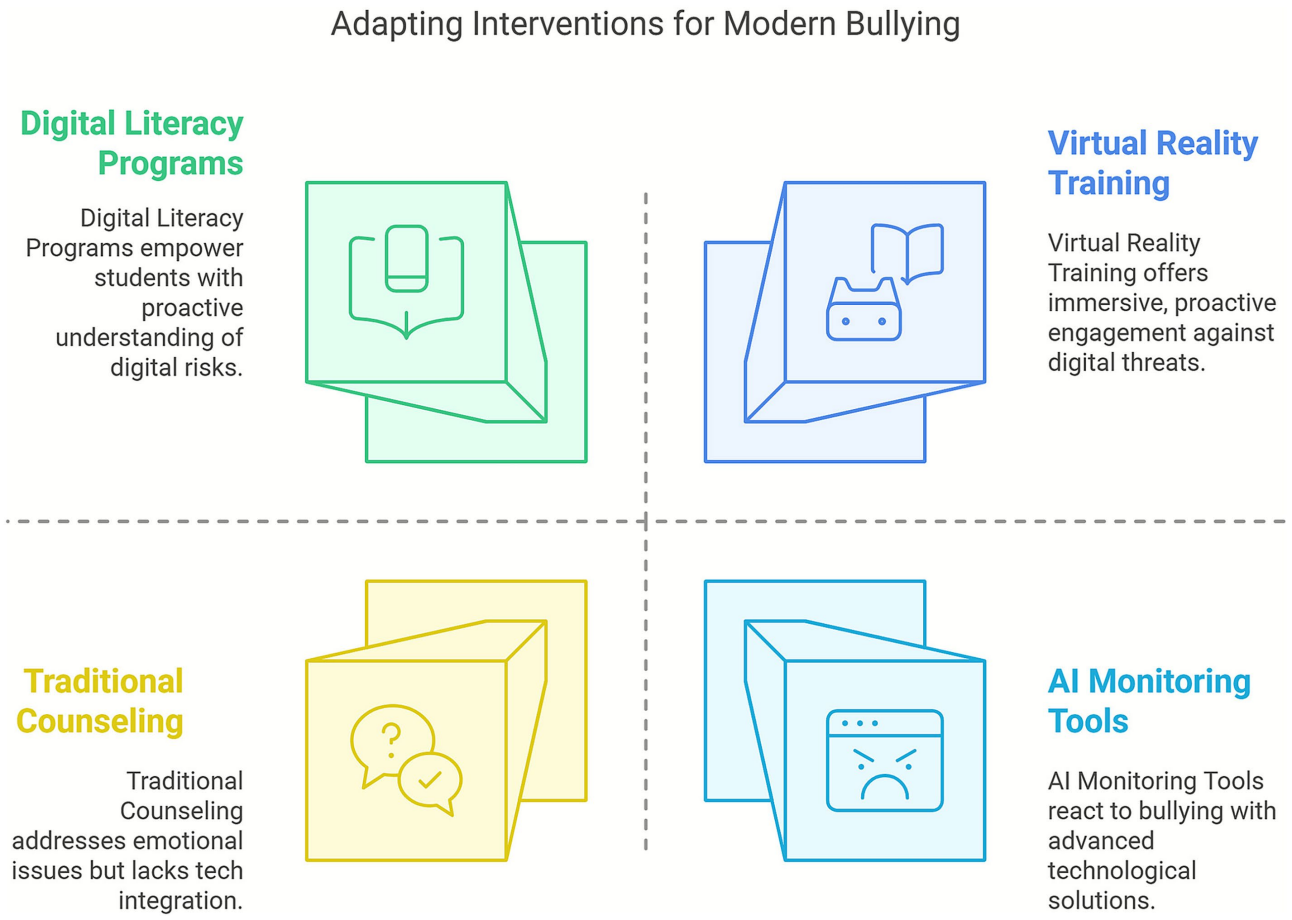
Adapting interventions for modern bullying.

To adapt effectively, psychological interventions must move beyond traditional face-to-face models and adopt interdisciplinary perspectives that integrate media psychology, computer science, and ethics. Rather than replacing human-centered approaches, AI tools should complement them—supporting early detection, tailoring emotional support, and helping school professionals respond proactively to new forms of harm. Thus, the future of bullying prevention depends not only on identifying risks, but on creating psychologically informed frameworks that evolve with digital threats (Figure 6).

## 4 Conclusion

The systematic review reveals that psychological interventions have significantly evolved over the last decade, transitioning from individual and behaviorist approaches to systemic models that integrate emotional, social, and cognitive dimensions (a). Among the various frameworks, cognitive-behavioral therapy (CBT) and social–emotional learning (SEL) stand out as the most effective in reducing bullying behaviors and supporting students’ emotional recovery (b). However, the success and sustainability of these interventions depend heavily on institutional commitment and the existence of clear educational policies that promote long-term implementation (c). Evidence also shows that such interventions not only enhance students’ emotional well-being but can positively influence academic engagement and performance (d). Finally, the emergence of cyberbullying and AI-mediated aggression underscores the urgency of developing new, proactive, and interdisciplinary responses that address the complexities of digital violence in contemporary school settings (e).

### 4.1 Limitations and future studies

This review is limited by its focus on studies published between 2015 and 2024, which may exclude earlier foundational contributions to the field. Additionally, the scope was restricted to secondary school populations, potentially overlooking relevant evidence from primary and tertiary educational settings. The heterogeneity in research designs, sample characteristics, and intervention formats across the included studies posed further challenges for direct comparison and synthesis. A methodological constraint concerns the use of the term session in the search strategy; while this decision aligned with the objective of identifying structured psychological interventions, it may have excluded programs labeled as training, workshops, or general school-based initiatives. Future reviews should adopt a more inclusive search syntax—such as (program OR intervention OR training OR workshop) AND bull AND (school OR education)*—to enhance comprehensiveness. Another limitation is the deliberate restriction to Open Access publications, which was implemented to ensure full transparency, replicability, and unrestricted verification of the dataset through its public release on Zenodo (DOI: 10.5281/zenodo.15091482). However, this choice may introduce publication bias by excluding high-quality paywalled studies. Finally, the rapid emergence of new forms of bullying, especially cyberbullying and AI-driven aggression, highlights the need for longitudinal and cross-cultural research that examines how psychological interventions can be adapted to evolving digital contexts.

## Data Availability

The datasets presented in this study can be found in online repositories. The names of the repository/repositories and accession number(s) can be found in the article/supplementary material.
